# FLED-Block: Federated Learning Ensembled Deep Learning Blockchain Model for COVID-19 Prediction

**DOI:** 10.3389/fpubh.2022.892499

**Published:** 2022-06-17

**Authors:** R. Durga, E. Poovammal

**Affiliations:** Department of Computing Technologies, School of Computing, SRM Institute of Science and Technology, Chennai, India

**Keywords:** image processing, artificial intelligence, extreme learning machine, privacy preservation, capsule learning model

## Abstract

With the SARS-CoV-2's exponential growth, intelligent and constructive practice is required to diagnose the COVID-19. The rapid spread of the virus and the shortage of reliable testing models are considered major issues in detecting COVID-19. This problem remains the peak burden for clinicians. With the advent of artificial intelligence (AI) in image processing, the burden of diagnosing the COVID-19 cases has been reduced to acceptable thresholds. But traditional AI techniques often require centralized data storage and training for the predictive model development which increases the computational complexity. The real-world challenge is to exchange data globally across hospitals while also taking into account of the organizations' privacy concerns. Collaborative model development and privacy protection are critical considerations while training a global deep learning model. To address these challenges, this paper proposes a novel framework based on blockchain and the federated learning model. The federated learning model takes care of reduced complexity, and blockchain helps in distributed data with privacy maintained. More precisely, the proposed federated learning ensembled deep five learning blockchain model (FLED-Block) framework collects the data from the different medical healthcare centers, develops the model with the hybrid capsule learning network, and performs the prediction accurately, while preserving the privacy and shares among authorized persons. Extensive experimentation has been carried out using the lung CT images and compared the performance of the proposed model with the existing VGG-16 and 19, Alexnets, Resnets-50 and 100, Inception V3, Densenets-121, 119, and 150, Mobilenets, SegCaps in terms of accuracy (98.2%), precision (97.3%), recall (96.5%), specificity (33.5%), and F1-score (97%) in predicting the COVID-19 with effectively preserving the privacy of the data among the heterogeneous users.

## Introduction

The epidemic of COVID-19 is believed to be one of the most hazardous illnesses that have a devastating effect on the lives of many individuals. Serious acute respiratory syndrome coronavirus (SARS-CoV) affects a huge population (1–3). India has the 2nd highest number of confirmed victims worldwide with 33,678,785 recorded positive cases and the third-highest number of COVID-19 fatalities (4, 5). As soon as, the count of COVID-19 infections rose to unimaginable heights, leaving the government and doctors ill-equipped to deal with them (6, 7). As a result, clinicians were having difficulty in identifying individuals who are COVID-19-positive due to a lack of testing models (8). The diagnosis of COVID-19 disorders relies heavily on clinical symptoms, epidemiological history, computed tomography (CT), and pathogenic testing. Most of the patients with COVID-19 show the same visual symptoms on CT scans as the other lung disorders (9–13) despite the use of a variety of radiological modalities. Symptoms of COVID-19 vary widely across individuals, and the disease is growing at an alarming rate (14).

As a result, hospitals may communicate information on patients with COVID-19 to get an appropriate diagnosis. It is a difficult challenge to securely share data (without exposing the privacy of users) and train a global model to recognize positive cases. As a result, current research is unable to exchange data with each other and train the model correctly. AI-based solutions are hindered by the difficulty of obtaining data from a variety of sources. Because healthcare facilities lack a privacy-preserving methodology, such sensitive information cannot be made available (15).

Currently, various artificial intelligence (AI) techniques are under exploration to solve the ambiguity in diagnosis (16–23). The existing AI techniques often require large data from a single source to train the deep learning model for a more accurate prediction. In contrast, data from a single source lack the feature distribution variance problem (24) that leads to the high misclassification rate affecting the diagnosis outcomes in terms of accuracy. These data unavailability problems can be reduced if many hospitals can share the data. But security and privacy concerns restrict the hospitals to share the data for research even. Hence, the existing AI methods need to be improved (25, 26), so that they can focus on collaborative learning while maintaining privacy.

### Motivation

According to the most recent WHO study, COVID-19 is an infectious illness that mostly affects the lungs, giving them a honeycomb-like appearance. Some people who have recovered from COVID-19 are left with long-term lung impairment. The primary goal of our study was to classify the patterns in the lungs caused by COVID-19, so that experienced radiologists do not overlook infection. Second, sharing the information to train a stronger deep learning model while keeping the privacy concerns of data providers (27). A deep learning-based model for automated identification of COVID-19 may be developed for sharing the data.

The first challenge is that owing to a lack of privacy, personal information cannot be made available. The second challenge is to use a blockchain network to train the global model (the Federated model). The third issue is difficult to obtain enough training data and improve the prediction model, which has an impact on the diagnosis ratio. Finally, recognizing the patterns of COVID-19 lung screening is a difficult process.

This problem motivated the invention of a collaborative deep learning model that can diagnose COVID-19 instances (28, 29) and share the results while ensuring the privacy and security of the hospitals (30). This paper proposes the FLED-Block framework, which learns jointly from various CT images acquired from various sources. The new capsule-deep extreme learning network is introduced in the suggested framework for enhanced segmentation and classification. A capsule network is developed for its specialization in recognizing abnormalities in medical images. To train a more accurate model using the previous approach, a large amount of data is required. The capsule network enhances the deep learning models' performance at the level of the models' internal layers. To improve classification accuracy and diagnosis, the suggested system combines the strong properties of capsule Networks and extreme learning machines (ELMs) to solve the issues. To better forecast COVID-19, ELM substitutes dense classification layers with capsule networks, which obtain strong feature maps. Privacy concerns are addressed using federated learning methods to distribute the learned model across any node in the network (31).

The main contribution of the paper

- The proposed algorithm detects and classifies the COVID-19 patterns from the CT images from multiple sources using an ensemble of capsule networks. The extreme learning machine in the capsule is used for better feature extraction to achieve better classification.
- This paper introduces the blockchain powered data collection and sharing unit, which collects the data from different heterogeneous sources and employs federated learning to maintain data privacy between the organizations with high-accuracy global model training.
- Finally, the excellence of the proposed algorithm is proved by experimenting with the different sources of datasets in which the performance metrics are evaluated and compared with other existing deep learning algorithms.

This research article proposed a blockchain empowered federated framework to improve the recognition of multiple-source heterogenous CT images and share the data among the hospitals while maintaining privacy and security. Also, ensembling of capsule networks and ELMs are used for effective feature extraction and classification to detect the COVID-19 among the different sources of publicly available heterogenous CT image datasets.

The paper is structured into four sections. The introduction is dealt in the first section and the second section presents the related works on the decentralized network. The data normalization, ensembled learning model, and blockchain-based federated data sharing mechanism are presented in Section FLED-Block Model. The experimentations, results, findings, and comparative analysis are discussed in Section Experimental Results. Finally, the paper is concluded in Section Results and Findings with future enhancement.

## Related Works

To detect the diseases as early as possible, Supriya et al. studied the trending medical imaging analysis techniques in terms of prediction, e-treatment, stage classifications, virtual monitoring, and data transmission (24). Various supervised learning classifiers such as SVM, DT, KNN, and ANN are utilized in medical imaging. Similarly, the blockchain is important for public access to medical data and data transfer across worldwide using numerous blocks in a distributed approach. The author concluded that current medical image transmission is ruled by these innovations in the recent days for serving the healthcare industry.

People who are tested for the COVID-19 virus may not get enough instructions on how to manage and decrease both the risk of infection and the spread of the virus. COVID-19 data cannot be widely disseminated because of concerns about patient's privacy. To address the restrictions outlined above, this article offers a privacy architecture based on federated learning and blockchain technology. The proposed infrastructure can improve public communication and provide alternate means of disseminating COVID-19 information. Furthermore, the suggested architecture may effectively address the problem of huge data silos and offer a shared model while respecting the privacy of data owners. It has also been determined that the planned infrastructure can withstand information security and privacy breaches (32).

An open-source software framework based on federated learning for medical imaging is developed by Georgios et al. as PriMIA which deals with multiple sources of pediatric radiology for classification purposes (33). The proposed PriMIA includes the deep convolutional neural network (DCNN) designed to categorize the various stages of cardiac disease using the trained pediatric chest X-ray image database. DCNN is trained with gradient-based model for inversion attacks that detect chest disease at the earliest. Shichang et al. developed the blockchain-based security model for detecting malicious nodes, by merging competing vote authentication techniques and aggregating strategies to a federated model. The key factor of this propounded model is to optimize the communication cost in transferring the big data during the federating learning process and avoided the two standard attacks called “free-riding attacks” and “model poisoning attacks” (34).

Kim et al. (35) suggested a “blockchain federated learning” (BlockFL) architecture for decentralized federated learning. The architecture named “BlockFL” was able to overcome the single point of failure by expanding its federated scope. To entrust the devices in public networks, the local training outcomes are included in the verification procedure. In terms of trust and motivation, Bao et al. (36) presented FLchain, a centralized, publicly audited, and healthy federated learning ecosystem. The traditional federated learning central coordinator is replaced by blockchain in FLchain.

The blockchain architecture with global models is learned using the concept of channels. The channel-specific ledger-based blockchain architecture was presented by Majeed and Seon (37). Each local parameter of the model is kept as blocks in a channel-specific ledger and followed the decentralized data maintaining procedure. Martinez et al. (38) use a crypto currency and federated learning to solve the issues of data privacy and safety. The authors suggest a detailed methodology, an off-chain record database for scalable gradient recording and reward. Abdul Salam et al. proposed a new federated learning algorithm especially for patients with COVID-19. The proposed neural network is pre-trained with chest X-ray (CXR) images of abnormal patients to predict the death severity and to provide the e-treatment. The limitation of the developed predictor is less sufficient for big data (39).

A model based on capsule networks (COVID-CAPS) was presented to address the limitations of CNN-based models while dealing with short datasets by Parnian et al. (40). This model may be used to detect COVID-19-positive cases from X-ray images. It was observed that the COVID-CAPS model performed better than that of the conventional network when the model parameters were adjusted.

Using the neural architecture search (NAS) algorithm and a federated NAS (FedNAS) algorithm, He et al. (41) proposed an experimental study on automated federated learning (AutoFL), which aims to improve the quality and productivity of local machine learning models that connect their model updates. For non-IID (i.e., not unique ID) clients, they found that the default parameters of local machine learning models did not suit the federated context.

## FLED-Block Model

**Figure 1:**
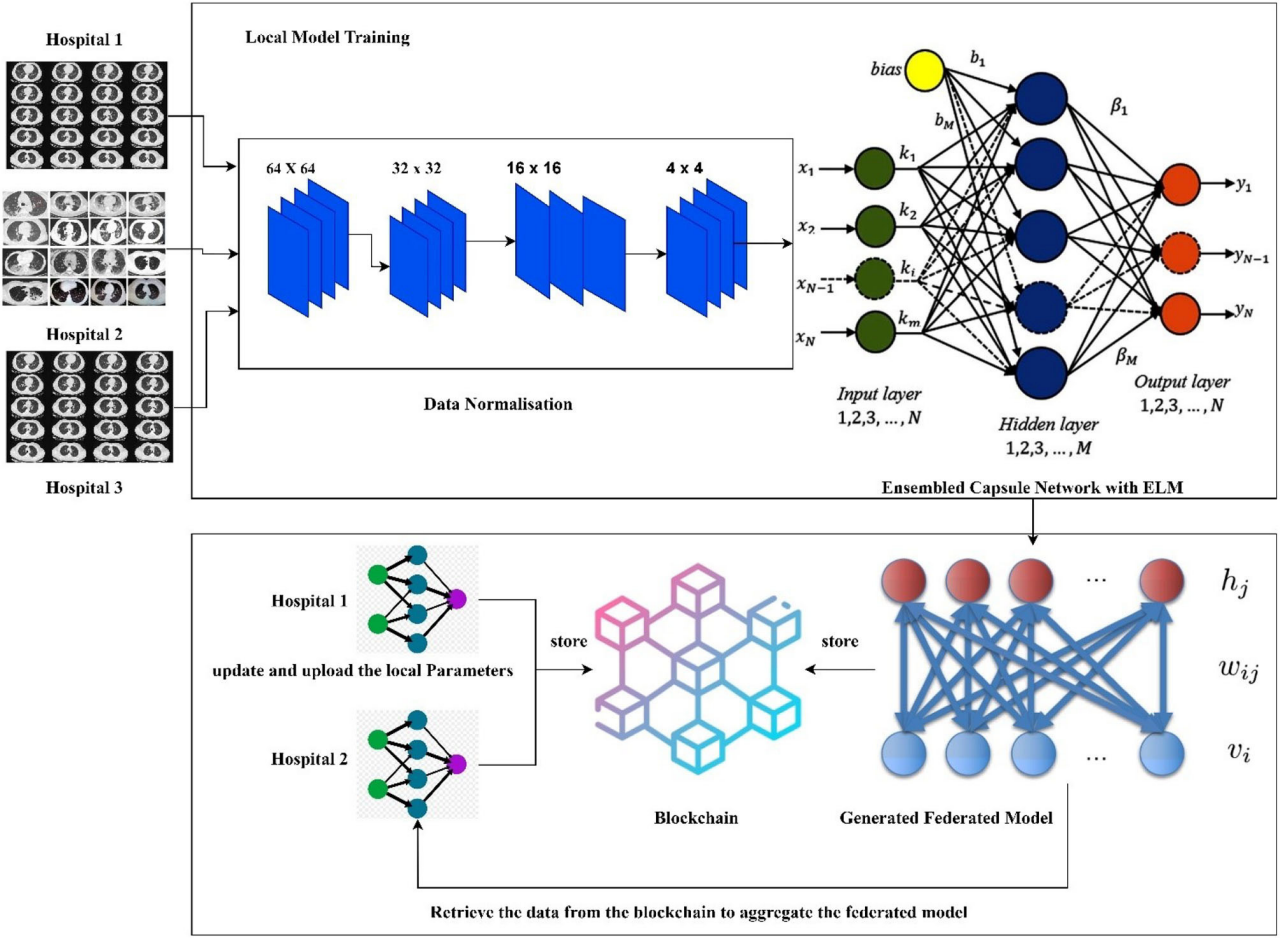
Proposed framework of the FLED-Block.

The hospitals and other healthcare organizations are showing a susceptible response to sharing the patients' data with other centers due to the breach of privacy of the patients. On implementing the deep learning models for an effective diagnosis of diseases, a large amount of data is required. The high amount of data processing increases the time complexity and leads to performance degradation most of the times. To handle real-time problems, this research proposes the new model FLED-Block, which can train and share the global models. Figure 1 presents the proposed framework.

The proposed FLED-Block architecture collects data cooperatively from various hospitals with a variety of different CT scanner types. As a first step in the process of data normalization, spatial normalization and signal normalization are being used. Then, deep learning algorithms are utilized to identify COVID-19 patterns in lung CT images. Image segmentation and training are done using the ensembled capsule network for greater generalization. As compared to other learning models, ensembled capsule network performed better.

The COVID-19 images are then classified using the extreme learning machines (ELMs). ELM is a neural network with one hidden layer which can self-tune. Finally, we employ the federated learning technique to build the global model and tackle the privacy issue. It gathers data, trains an adaptive model together, and then distributes this model throughout the public network. However, federated learning enables hospitals to keep their patient's information private while exchanging just weights and gradients *via* blockchain technology. Data from different hospitals may be communicated safely and securely using a decentralized architecture without compromising the security of patient's information.

More precisely, the proposed framework (1) collects the data from the different sources (medical health care centers), (2) trains the model by the hybrid capsule learning network for segmentation and classification of COVID-19 images, and (3) collaboratively shares the hybrid model using blockchain with federated learning while preserving the privacy of the organization.

### Materials and Methodologies

The role of artificial intelligence has occupied an unavoidable position in clinical diagnosis (42). Additionally, the deep learning algorithms need to be trained with the huge amount of data, and data collection is considered to be more important for validating the proposed model (43). The different heterogeneous CT image data sources were used for training the proposed model. The description of the datasets used for evaluating the model is presented as datasets 1, 2, and 3.

#### Dataset-1

**Figure 2:**
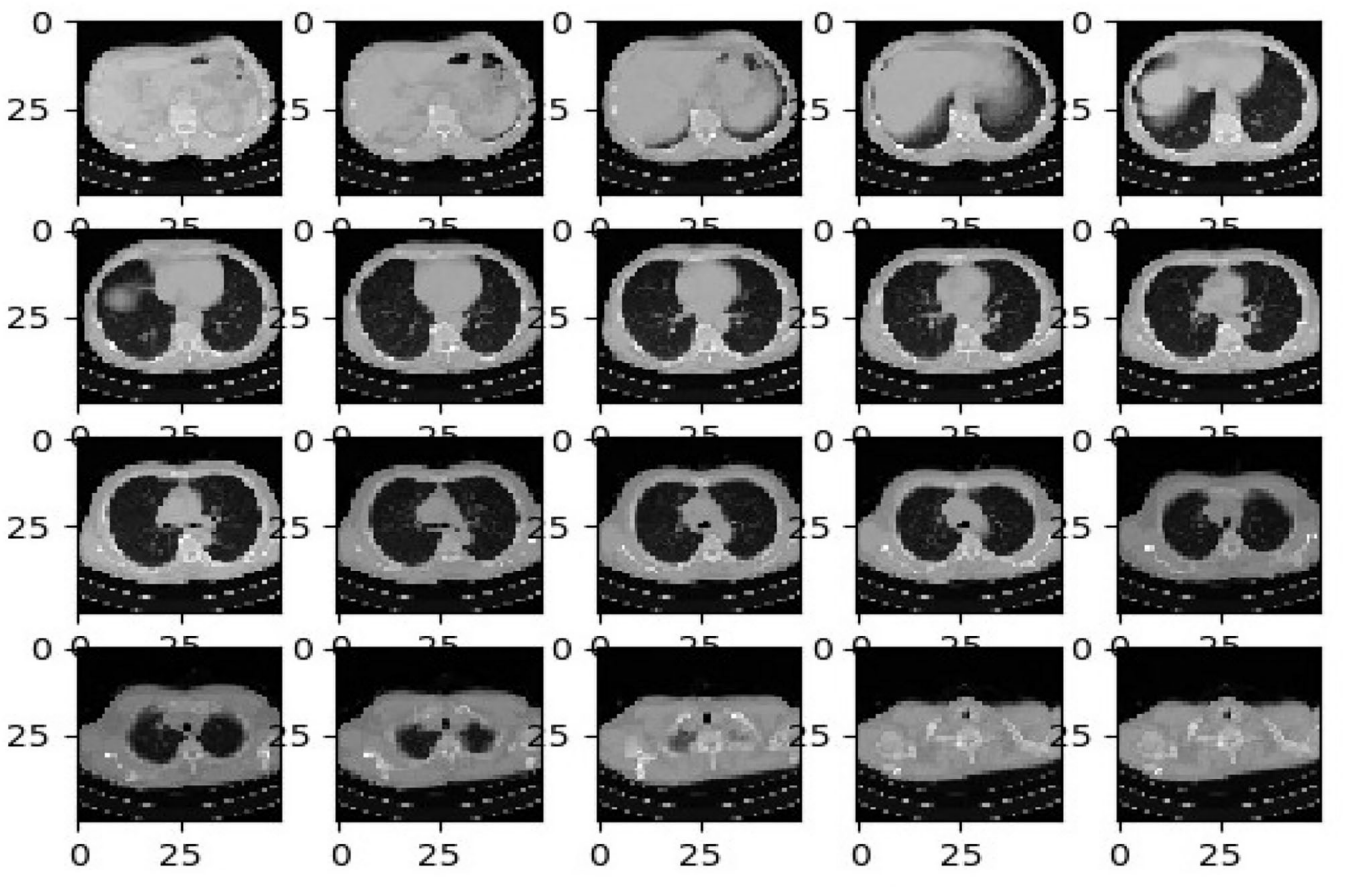
Computed tomography specimen images—Dataset-1.

The first dataset holds 34,006 CT scan slices from three hospitals by 89 individuals, with 28,395 of the CT scan slices belonging to patients with COVID-19-positive (44). A total of six separate scanners have scanned the data, which includes the CT scan slices for 89 distinct people. In all, 68 of the 89 participants tested positive for the COVID-19 virus, whereas the other 21 tested negative. The gathered CT image datasets are shown graphically in the below table. Experimentation utilizing image datasets is shown in Figure 2.

#### Dataset-2

The second dataset is made up of patients with confirmed COVID-19 infections who have unenhanced chest CTs (45). “Hypertension or coronary heart disease, diabetes, and interstitial pneumonia or emphysema” were the most common concomitant disorders mentioned by patients. An in-patient setting was used to collect these images from patients who had positive “Reverse Transcription Polymerase Chain Reaction” (RT-PCR) tests for COVID-19 and accompanying clinical symptoms between March 2020 and January 2021. The images were taken during this period. No intravenous contrast was used during the CT examinations, which were performed in “Helical” mode on a NeuViz 16-slice CT scanner (Neu soft medical systems). All images are in DICOM format and are composed of 512 X 512 pixel 16-bit grayscale images.

#### Dataset-3

In this dataset 3, there are 349 COVID-19 CT scans from 216 patients and 463 non-COVID-19 CT images. These datasets comprised of initial images and both datasets were taken at the point of care in an epidemic situation from patients having RT-PCR confirmation for the presence of SARS-CoV-2. In (46, 47), the datasets are described in more detail.

**Figure 3:**
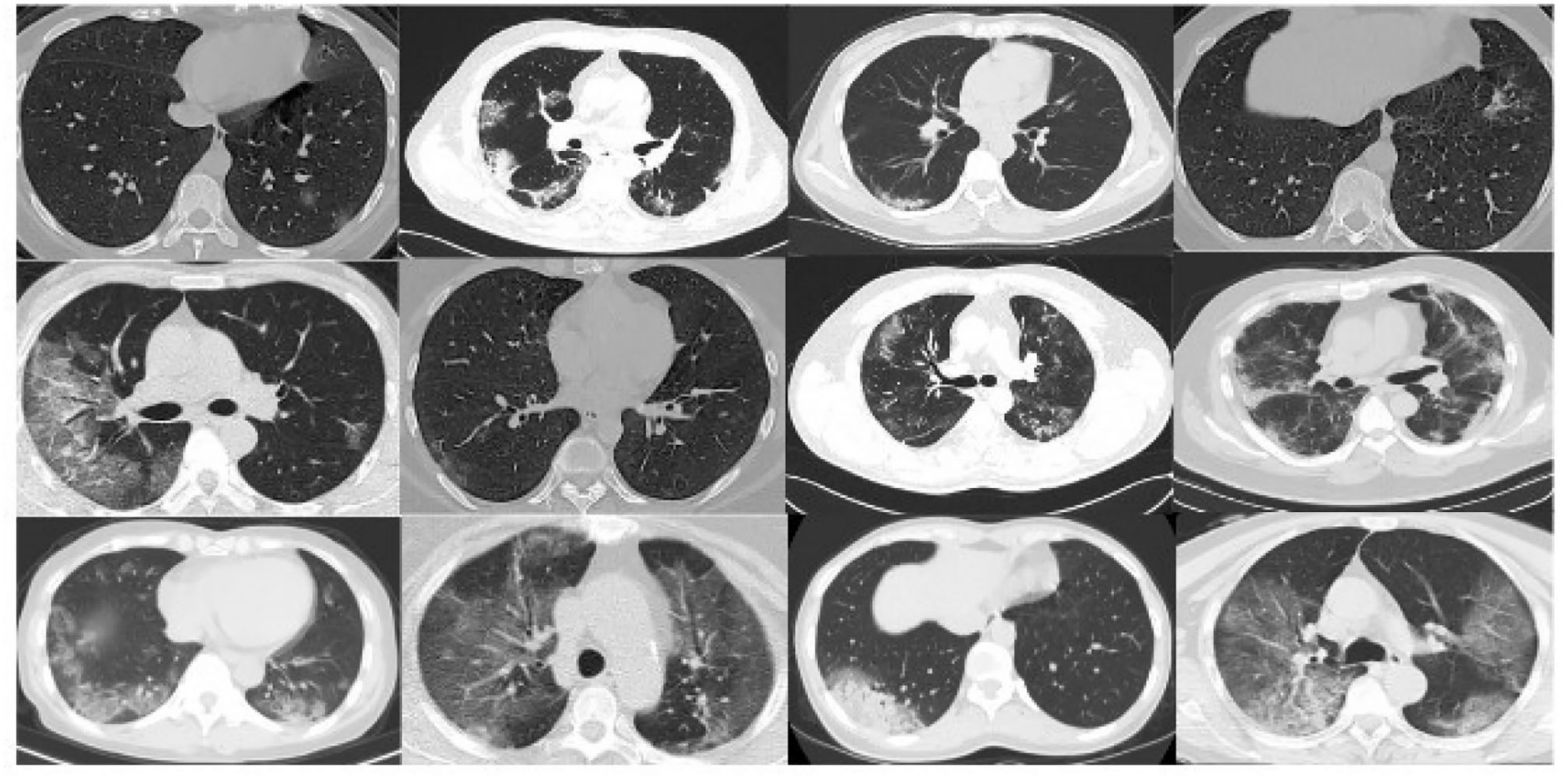
Computed tomography specimen images—Datasets 2 & 3.

As the data are acquired from heterogeneous data sources, we develop the normalizing approach to adopt the CT scan images for a federated learning mechanism. Table 1 gives an overview of the datasets obtained for assessing the proposed federated learning model. Figure 3 illustrates the sample datasets utilized for experimentation.

**Table 1:** Summary of the dataset details used for the proposed research.

**Datasets**	**Source of datasets**	**No of COVID-19 patients**	**No of Non-COVID-19 infections**	**Image formats**	**Number of patients**	**Total number of CT scan images**
Datasets-1	CC-19 datasets [ ]	63	26	CT Scan images	89	34,006
Datasets-2	COVID-19-CT datasets	783	217	DICOM Images	1,000	45,002
Dataset-3	COVID-19 CT datasets [ ]	216	463	CT Scan Images/DICOM	689	3,490

## Data Normalization

The data normalization technique mentioned in Krizhevsky et al. (48) is adopted by the proposed research. Since the heterogeneous data were used, a strong normalization technique is required to enhance the performance of the proposed federated learning models. As mentioned in Krizhevsky et al. (48), two types of normalization such as signal normalization and spatial normalization techniques are used in handling the CT scan images. The functionalities of these normalization techniques are discussed as the signal normalization technique and spatial normalization technique.

### Signal Normalization Technique

Signal normalization calculates with the voxel's intensity based on the lung window. As every CT scan has Hounsfield units (HU), two types of windows such as window level (WL) and window width (WW) are mostly used in medical practices. Based on this window size, the normalized value is calculated using equation 1.


(1) $$\begin{array}{l} \text{Onormalized}=\left(\text{O}-\text{ WL}\right)/\text{WW} \end{array}$$

Where Onormalized is the image to be normalized in terms of intensity and O is the input original image. For this experimentation, lower bound window size has been chosen in the range of [−0.05, 0.5].

### Spatial Normalization Technique

The spatial normalization is adopted based on the dimension and resolution of the CT scan images. All the CT scan images are converted to a standard resolution of 332 × 332 × 512 mm^3^ as mentioned in Krizhevsky et al. (48). This normalization technique is applied to all the collected datasets and all formats of images are normalized to standard format (49) which can be applied to federated learning. It achieves better learning and performance.

## Ensembled Capsule-Based Model Training

Recently, usage of the deep learning framework has gained its own popularity with the strong feature extraction layers and its classification mechanism. Over the past few years, convolutional neural network (CNN) is predominantly utilized for image classification analysis. Since the pooling layers of CNN do not consider the spatial relationship between the features in an image, this leads to high computational complexity and even affects the classifier's performance. To achieve better classification accuracy and diagnosis, the proposed system ensembles the powerful features of capsule networks and extreme learning machines (ELM) to overcome these above-mentioned problems. In this approach, capsule networks are used for extracting the strong feature maps whereas ELM replaces the traditional dense classification layers for better prediction of COVID-19.

## Capsule Networks

**Figure 4:**
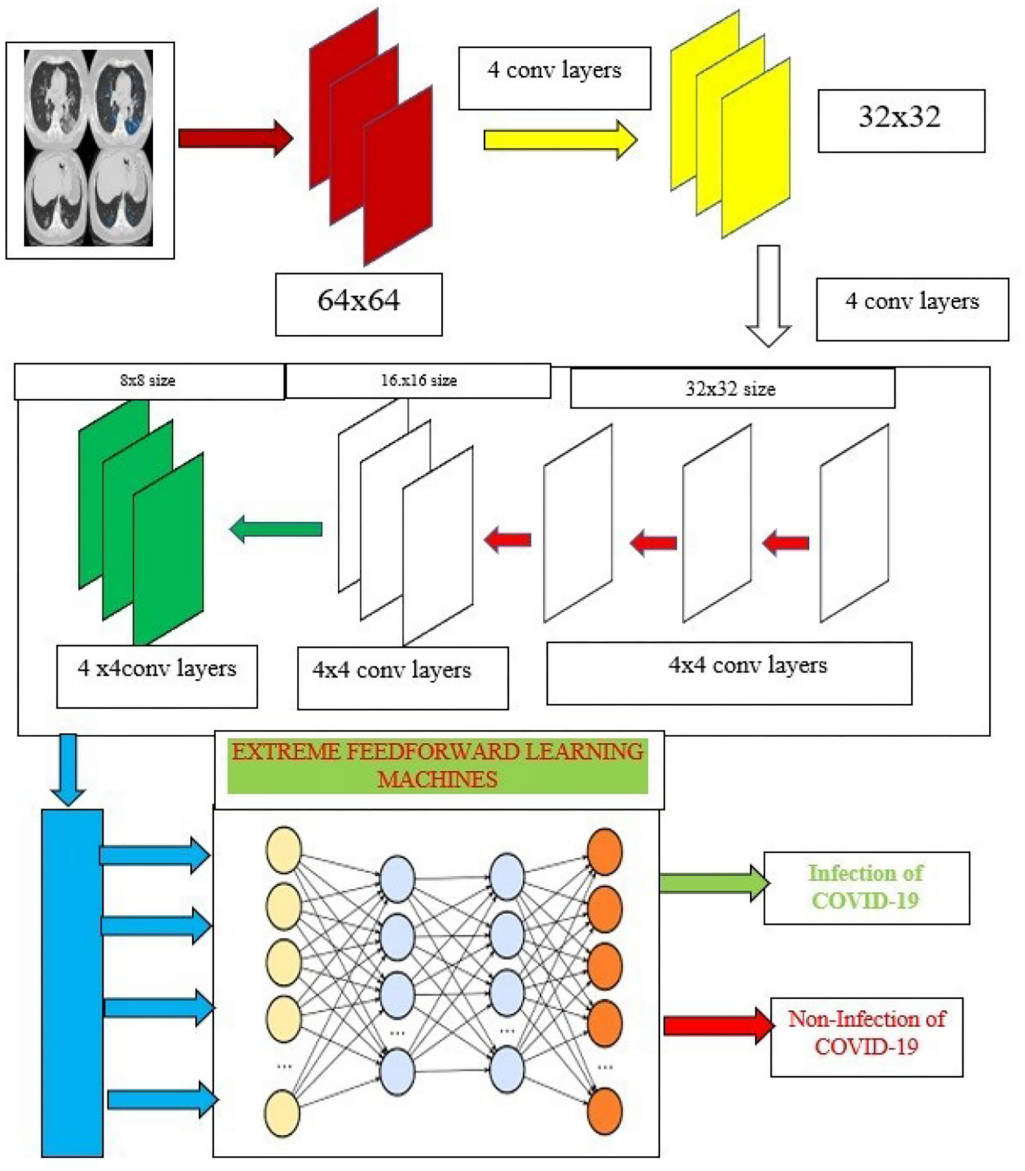
Capsule ensembled ELM layers for achieving the feature extraction and classification accuracy.

Capsule network (50) had “(1) convolutional layer, (2) hidden layer, (3) Primary Caps layer, and (4) DigitCaps layer” which had addressed the limitation. Figure 4 shows the complete architecture for the proposed training model. The input normalized image is given as the input to the proposed capsule networks. It is divided into two phases.

- Probability of existence in entities.
- Entities' instantiation parameters.

To encode the imperative spatial association between low- and high-level features within the image, equation 2 is calculated, whereas the input vectors “s” and the weight matrix “W” and component vector “U.”


(2) $$\begin{array}{l} Y\left(i.j\right)=W\text{\_}\left(i,j\right)U\left(i,j\right)\;*\;S\text{\_}j \end{array}$$

The sum of the weighted input vectors is calculated to determine the current capsule “D” using the equation 3


(3) $$\begin{array}{l} S\left(j\right)=\underset{j}{\sum }Y\left(i,j\right)\;*\;D\left(j\right) \end{array}$$

Finally, non-linearity is applied using the squash function using equation 4.


(4) $$\begin{array}{l} Y\left(i.j\right)=W_{i,j\;}U\left(i,j\right)\;*\;S_{j} \end{array}$$

The distribution of the “low level capsule to the high-level capsule” is progressively adjusted according to the outcome until an optimal distribution will be attained.

## Extreme Learning Machines

The proposed research incorporates the capsule networks for better feature extraction which can help to achieve the highest accuracy of classification. Additionally, these features are then fed to the extreme learning machines (ELMs) which are used to classify the images. ELM is a kind of neural network that utilizes the single hidden layers and works on the principle of autotuning property which is depicted in Figure 4.

Extreme learning machine exhibits better performance when compared to the other learning models. Performance metrics considered are high speed and less computational overhead. The learning models compared are support vector machines (SVMs), Bayesian classifier (BC), K-nearest neighborhood (KNN), and even random forest (RF) (51).

The neural network utilizes a single hidden layer, that does not require the tuning mandatorily. ELM uses the kernel function to yield good accuracy for better performance. The major advantages of the ELM are minimal training error and better approximation, since ELM uses the autotuning of the weight biases and non-zero activation functions. The detailed working mechanism of the ELM is discussed in Huang et al. (52) and Wang et al. (53).

Mathematically, the characteristic of ELM is represented by


(5) $$\begin{array}{l} {\text{f}}_{\text{L}}\left(\text{x}\right)=\sum_{\text{i}=1}^{\text{L}}{\text{β}}_{\text{i}}{\text{h}}_{\text{i}}\left(\text{x}\right)=\text{h}(\text{x})\text{β} \end{array}$$

where x → input

β → output weight vector and it is given as follows as:


(6) $$\begin{array}{l} \beta =(\beta \_1,\beta \_2,\ldots \ldots \ldots \ldots .\beta \_L{]}^{\wedge }T \end{array}$$

h(x) output hidden layer which is given by the equation 7


(7) $$\begin{array}{l} \text{h}\left(\text{x}\right)=[{\text{h}}_{1}\left(\text{x}\right),{\text{h}}_{2}\left(\text{x}\right),\ldots \ldots \ldots \ldots ..{\text{h}}_{\text{L}}\left(\text{x}\right) \end{array}$$

- Hence, the outcome can be found by the equation 8.


(8) $$\begin{array}{l} f_{L}\;\left(\text{x}\right)=\;\text{h(x)}\beta =\text{h(x) }H^{\wedge }T\;{\left(1/C\;HH^{\wedge }T\;\right)}^{\wedge }\left(-1\right)\;O \end{array}$$

where O is output target vectors which are solved by bias weights of hidden layers which are solved by the Moore's pseudo-concepts (52). Based on equation (7), the infectious impact of COVID-19 on lungs is classified effectively. The working mechanism of the propounded network is presented in Algorithm 1.

**Algorithm 1:** Pseudo code for the proposed ensembled algorithm.

1 **Inputs:** Normalized Input Images: I
2 **Output:** Presence of COVID-19 diseases on Lungs
3 For *n* = 0 to Max_iteartions *n* = No of iterations
4 Features F = Capsule(I)//Using
Equations (2–4)
5 Output Function= ELM(F)//Using
Equation 7
6 If Output = = threshold//User-based threshold
7 COVID-19 is detected
8 Else
9 Normal Condition is detected
10 End
11 End
12 End

## Federated Learning for Global Training

**Figure 5:**
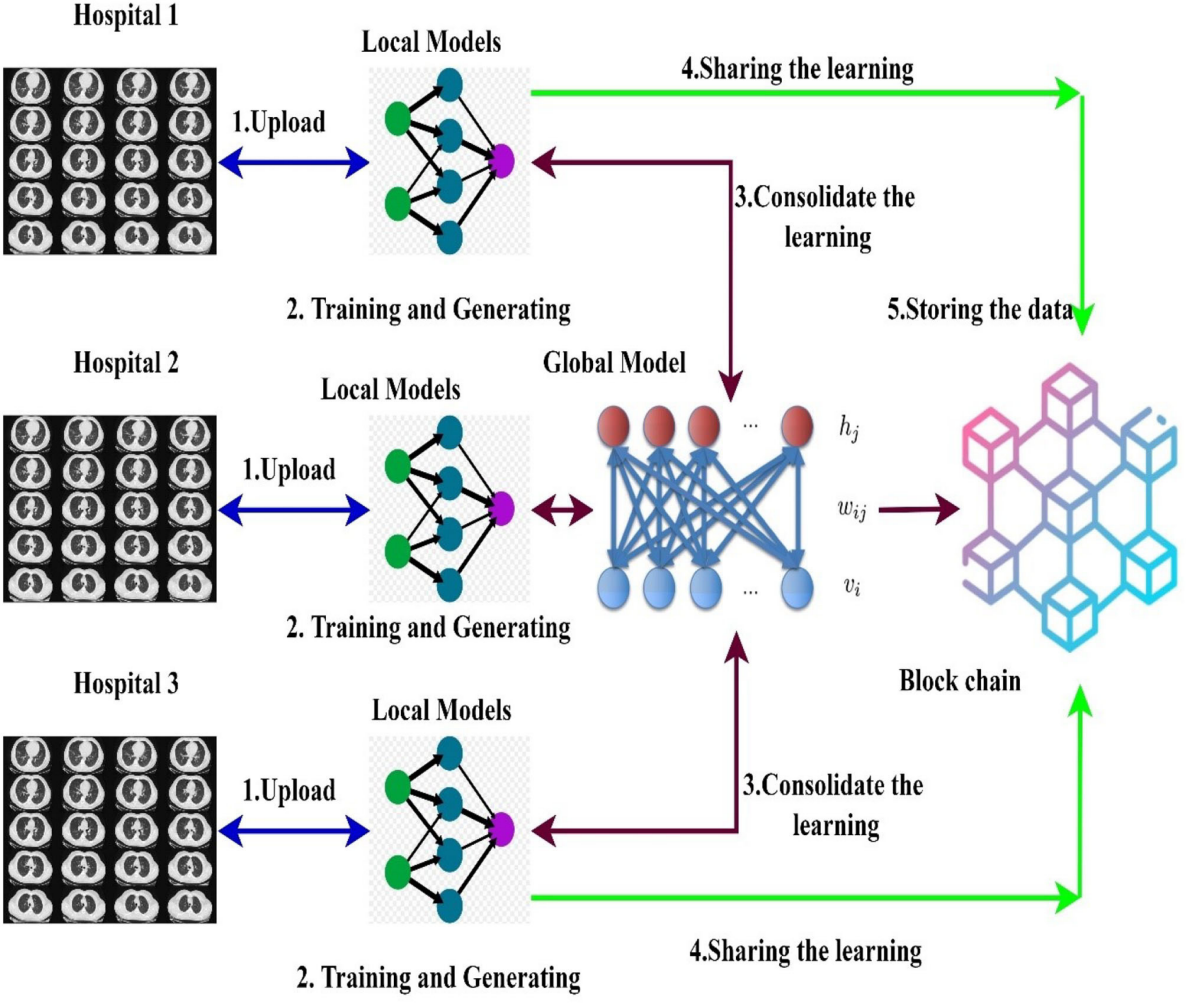
Blockchain empowered federated learning models used in the proposed framework.

In this section, decentralized data sharing mechanism with multiple hospitals has been considered. The proposed model adopts federated learning for sharing the hospital's models without sacrificing privacy and also aggregates the different models shared by the different hospitals. For this scenario, we consider the number of hospitals as H and d as the overall datasets. In the proposed federated model, the ensembled learning model is considered as the global model M in which the weights W of the ELM are distributed randomly to the other hospitals. Figure 5 shows the federated learning model adopted in the proposed research.

A blockchain-based federated learning framework that can be used to train and share in a collaborative way (50). The hospital uses a global or collaborative model, which is referred to as “federated learning,” to integrate the weights of the locally trained model (54). Initially, the data are gathered from many sources into the local model devised with a normalization approach to cope with various types of CT scan data. After normalizing the data, the second step is to segment the images using the ensembled capsule network to train the model to recognize COVID-19 suspects. Finally, we distribute the weights of the local model across the blockchain network to train a global model.

Mathematically, let H be the number of hospitals, d be the total datasets which consists of training and testing datasets as in equation 8


(9) $$\begin{array}{l} D_{i}^{train}=\left\{\left(X_{i,j}^{train},Y_{i,j}^{train}\right)\right\}where\;j=1\;to\;N-train\;data \end{array}$$

Also testing data are represented as in equation 9


(10) $$\begin{array}{l} D_{i}^{test}=\left\{\left(X_{i,j}^{test},Y_{i,j}^{test}\right)\right\}where\;j=1\;to\;N-test\;data \end{array}$$

Hence, the total dataset used for training the global model is in equation 10.


(11) $$\begin{array}{l} \text{D}\left(\text{i}\right)=\;D_{i}^{train\;}\;UD_{i}^{test} \end{array}$$

Since the data D(i) are collected from the heterogeneous source of hospitals, the distribution of data remains unequal. In every round of the communication, weights W of ELM are distributed among the hospitals. The hospitals create the local model with weights obtained and stored in the blockchain network. Then, weights are updated and loaded into the blockchain network for each round. The updated weights are mathematically updated like in equation 11


(12) $$\begin{array}{l} \eta ={\text{W}}^{\text{i}}-{\text{W}}^{\text{l}} \end{array}$$

where Wi and Wl are considered as distributed weights of the global model and weights of the local model, respectively. Finally, the all-local models in the blockchain are aggregated to form the new learning model which works on the principle of ELM which is implemented in the proposed deep learning algorithm.

## Blockchain Framework for Federated Learning

The proposed framework incorporates the blockchain architecture for an effective and secured data retrieval and sharing process of federated learning. Multiple hospitals can collaboratively learn and train the models for the better detection of diseases. Based on the multiple-organization blockchain architecture designed by (55, 56), the proposed work discusses about the data retrieval and sharing process adopted in the proposed approach.

### Blockchain-Based Data Retrieval Process

Every hospital provides the data (local model) and stores it as a transaction in the block chain network (57). Retrieving the data from the nodes depends on the two parameters such as distance between the nodes(d) and ID of the hospital (ID). Based on the distance between the hospitals, a unique ID is created. The blockchain maintains the log tables to store the unique ID of the user hospital. The data are retrieved from the neighborhood hospitals identified by their unique IDs.

Mathematically, hospitals are denoted by X partitioned as the different communities in which the hospitals are considered as nodes. The expression used to measure the neighborhood distance between the nodes is given by equation 12


(13) $$\begin{array}{l} \begin{array}{c} d\left(X\left(i\right),X\left(j\right)\right)=\sum_{p,q,\in }\left\{X\left(i\right)UX\left(j\right)-X\left(i\right)nX\left(j\right)\right\}\\ Attributes\;of\;the\;Nodes/\sum_{p,q,\in }\left\{X\left(i\right)UX\left(j\right)\right\}\;*\\ Attributes\;of\;Nodes\;*\;\log\left(X\left(i\right),X\left(j\right)\right) \end{array} \end{array}$$

where X(i) and X(j) are the neighborhood hospitals located at i^th^ and j^th^ position which are differentiated by their unique ID.

In the next stage, the consensus process is used to train the FLED-Block model using the stored local models. In this way, all of the nodes work together to train the FLED-Block. It gives proof of work, which allows the data to be shared among the various nodes. The consensus approach verifies the quality of the local models during collaborative training by calculating the mean prediction accuracy error (MPAE). The MPAE is a result of a better ability to forecast the future. All data on the node are encrypted using high random Chaotic public (58) and private keys (50, 52, 53) to keep the data confidential. All transactions are subjected to MPAE, which is then recorded in the distributed ledger of the blockchain.

### Blockchain-Based Data Sharing Process

Security is a vital role in sharing the data between requester and source hospitals (59). Instead of sharing the complete data, hospitals can provide only learned models with the requesters. The hospitals can communicate with each other, and consensus algorithm is used to learn from federated data. The providers' and requesters' data stored in the blockchain nodes (60). To maintain data privacy, only learning models are shared instead of original data information (61). In the first phase, each hospital uploads the image datasets for collaboratively learning. In the second phase, hospitals share the locally trained model weights with the blockchain and use the federated learning to aggregate all the local models into global models.

## Experimental Results

The proposed model was developed using opensource TensorFlow federated version 2.1.0, whereas the classical models are developed using TensorFlow version 1.8. with both the models using Keras as backend. The complete experimentation is carried out on a PC workstation with Intel Xeon CPU, NVIDIA Titan GPU, 16GB RAM, and 3.5 GHZ operating frequency. Each dataset is splitted into 70% for training, 20% for testing, and 10% for validation, respectively. The 70% of all the three datasets (23,804, 31,501, and 2,443 images) are used for training the proposed model using TensorFlow federated version 2.1.0, and it is also used to train the other classical model using TensorFlow version 1.8. These models are implemented on the federated blockchain using python 3.9.1. The user interface for the blockchain runs on CSS script. This setup is utilized for validating and testing the algorithm. The proposed architecture is evaluated by the various performance metrics such as accuracy, precision, recall, specificity, and F1-score. These metrics are the representation of the model's ability that how correctly the model differentiates between the COVID-19 and non-COVID infections among the subjects using the proposed federated model and other classical models (62). Table 2 presents the mathematical expressions used for calculating the performance metrics.

**Table 2:** Comparative analysis of the different algorithms in detecting the COVID-19 using dataset 1.

**Algorithm**	**Performance metrics**				
	**Accuracy**	**Precision**	**Recall**	**Specificity**	**F1-Score**
VGG-16	0.8269	0.833	0.8234	0.170	0.832
VGG-19	0.833	0.843	0.823	0.173	0.840
Alexnets	0.834	0.823	0.814	0.189	0.826
Resnets-50	0.845	0.823	0.832	0.164	0.834
Resnets-100	0.849	0.843	0.834	0.167	0.838
Inception V3	0.80	0.82	0.821	0.190	0.801
Densenets-121	0.82	0.83	0.834	0.167	0.812
Desnsenet-119	0.78	0.793	0.80	0.200	0.80
Densenets-150	0.81	0.802	0.794	0.80	0.73
Mobilenets	0.782	0.784	0.778	0.783	0.778
SegCaps	0.89	0.934	0.923	0.07	0.930
Proposed model	0.982	0.973	0.965	0.0335	0.970

A medical diagnosis-based system needs to have “high accuracy, high precision and recall.” To solve the model's overfitting problem and improve the generalization problem, the early stopping method (63) is used. This method can be used to end the proposed network training. when the validation performance shows no improvement for N consecutive times.

## Results And Findings

**Figure 6:**
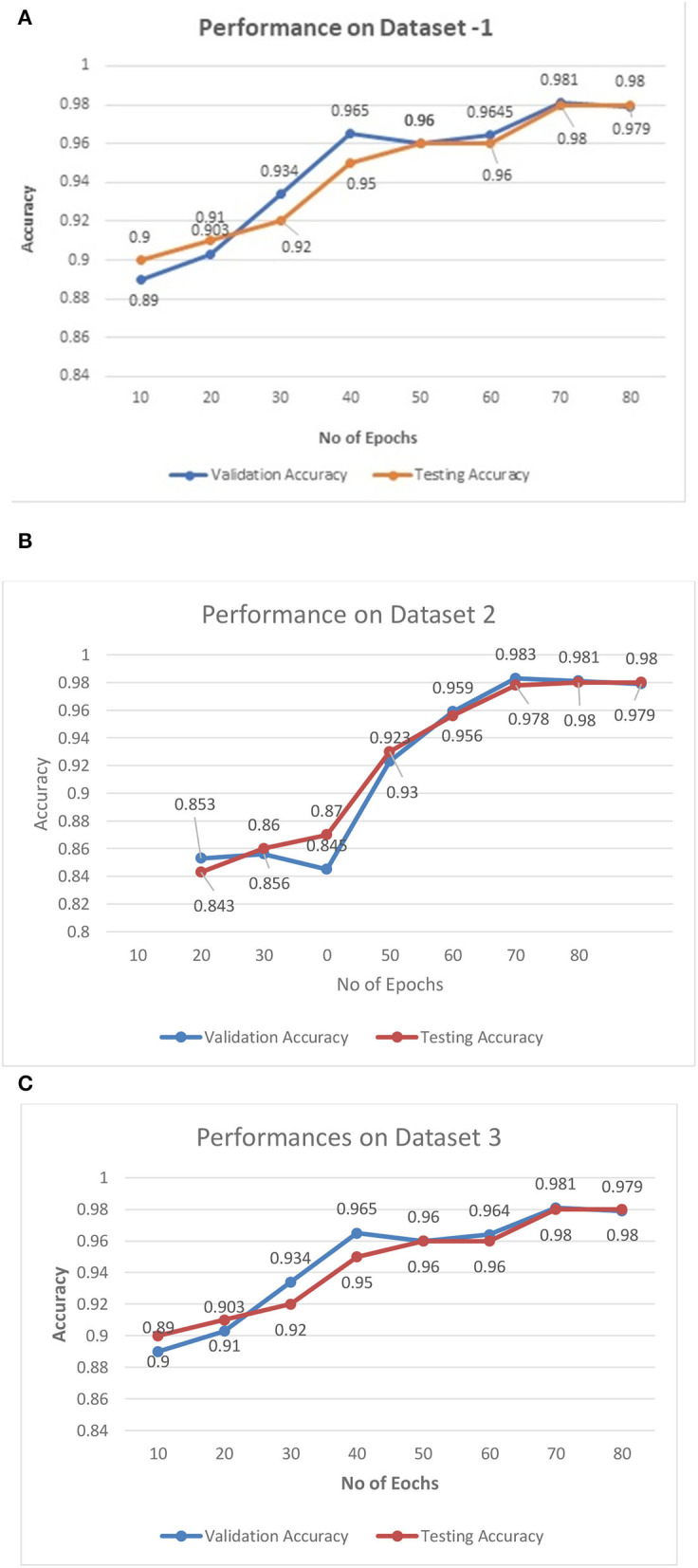
**(A–C)** Training—validation curves for the proposed algorithm for different datasets.

The performance of the proposed architecture is validated in three folds. In the first fold, the performance metrics of the proposed algorithm are calculated for the different CT datasets. Additionally, loss validation curves (LVC) are calculated for validating the performance of the proposed architecture. Finally, the excellence of the proposed algorithm has been proved by comparing it with the other existing deep learning algorithms. This section presents the performances of the proposed model using the different datasets as depicted in Figures 6A–C.

**Figure 7:**
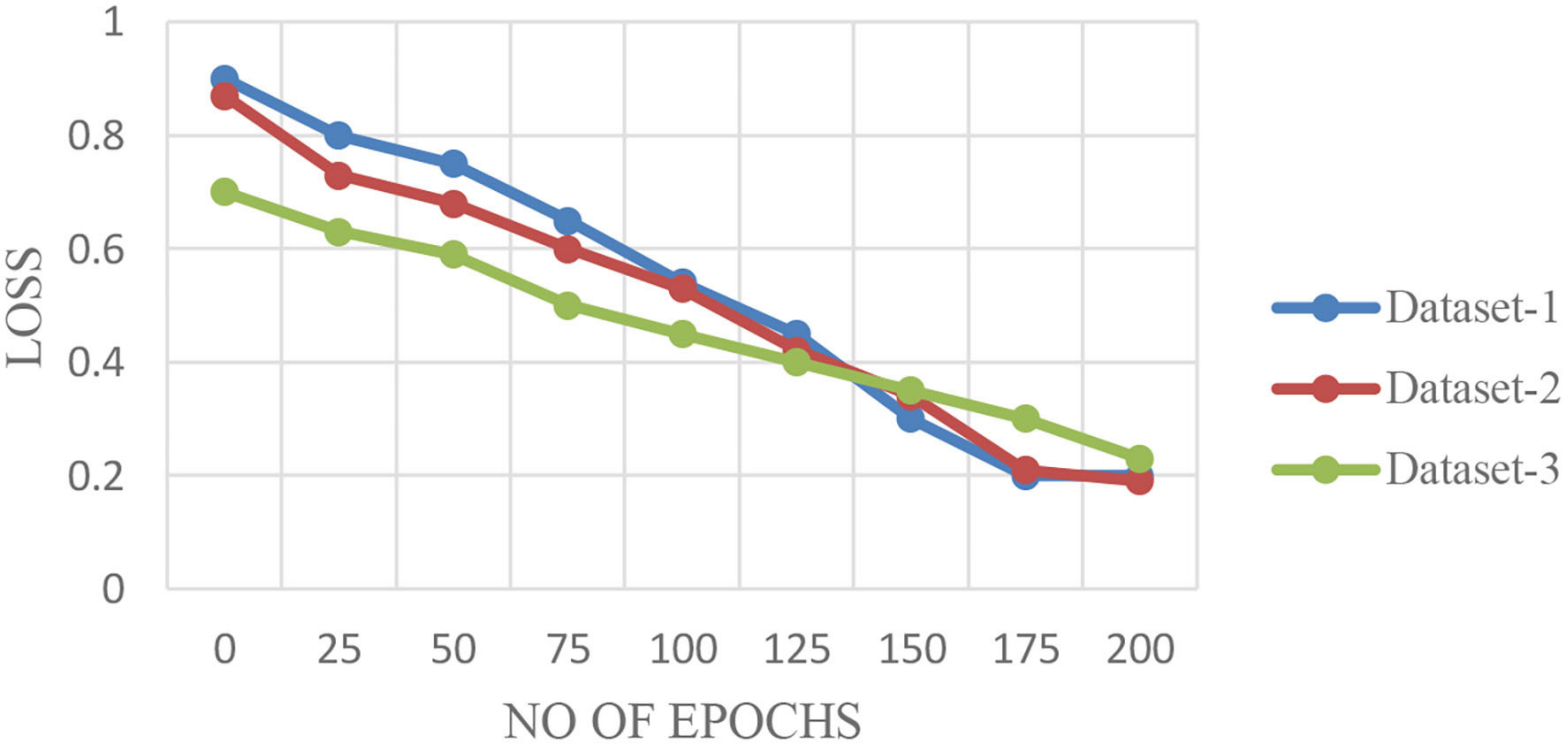
Loss validation curves for the proposed algorithm for different datasets.

Figures 6A–C illustrate the validation curves for training the proposed model with the different datasets whereas Figure 7 shows the loss validation curves of the model. From Figure 6A, it is found that the root mean square error (RMSE) between the training and validation model is found to be 0.001. A similar fashion of characteristics is found in the Figure 6C. As more datasets are involved in dataset 2, RMSE is a little higher in Figure 6B, i.e., RMSE is 0.0014. Hence, the average performance of the proposed model in detecting the COVID-19 is found to have 98.5 (dataset 1), 98.3 (dataset 2), and 98.5% (dataset 3), respectively.

**Figure 8:**
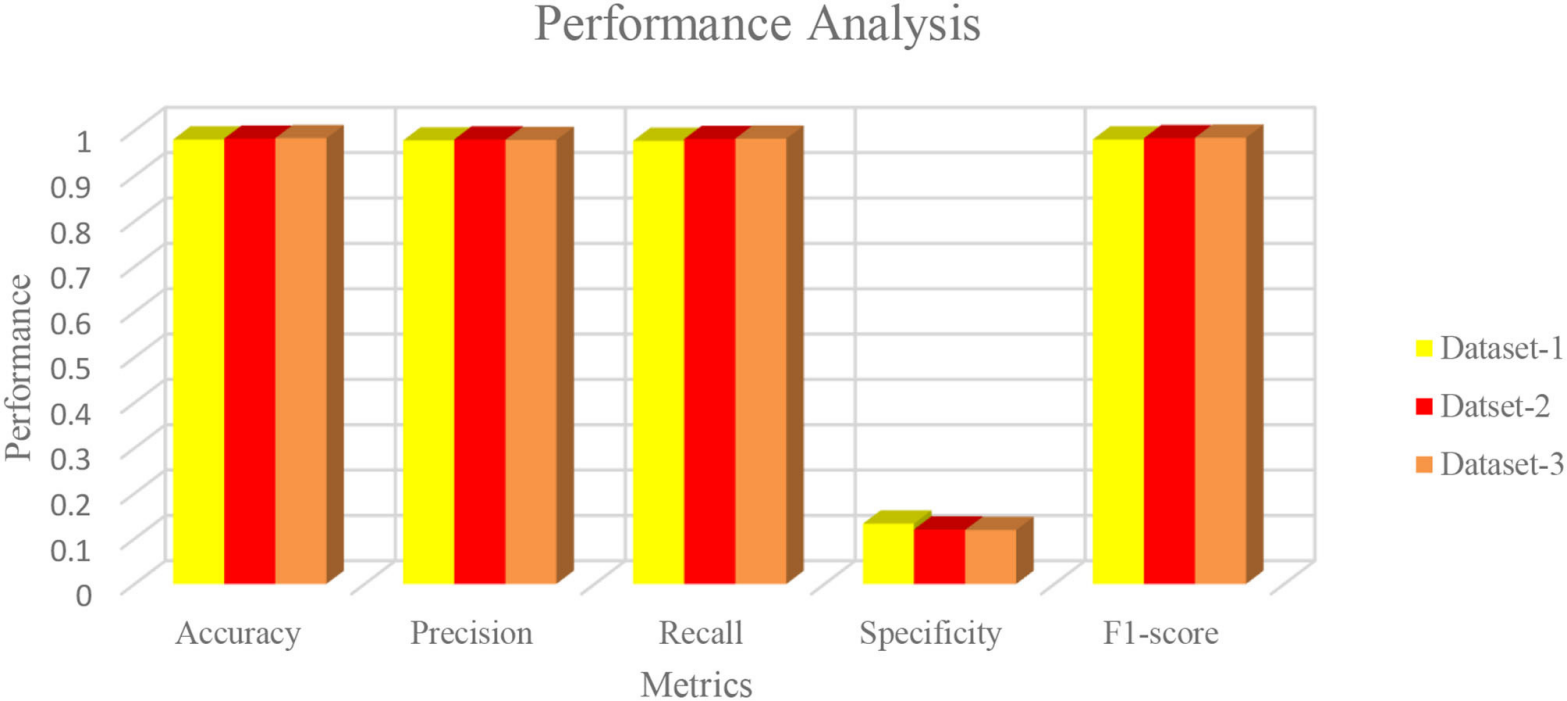
Performance metrics of the proposed algorithm with the different datasets.

The characteristics of capsule feature extraction and ELM as the classification layer in the proposed model help to maintain the uniform performance for the multi-source heterogeneous datasets. Figure 8 depicts the performance metrics of the propounded model in handling the different datasets. It is found that the average performance of the model ranges from 98.4 to 985% of accuracy, 97 to 98% of precision, and 97.5 to 98% of recall. Furthermore, it is found that the proposed model exhibits high false alarm rates and low specificity for all the three datasets as depicted in Figure 8.

To prove the excellence of the proposed model, a comprehensive comparative analysis between the deep learning algorithms such as VGG-16/VGG-19 (64–66), AlexNETS (67), DenseNETS (68–70), ResNETS-50/100 (59, 71), and even SegCAP (72) modules is done. Table 2 presents the performance of the different deep learning models and the proposed model in handling the dataset 1 From Table 2, it is found that the proposed model has shown the highest performance and topped over SegCAP networks and Resnets-100 which has produced the considerable good performances. Table 3 performs the divergent models with the proposed model.

**Table 3:** Comparative analysis of the different algorithms in detecting the COVID-19 using dataset 2.

**Algorithm**	**Performance metrics**				
	**Accuracy**	**Precision**	**Recall**	**Specificity**	**F1-Score**
VGG-16	0.797	0.783	0.7563	0.289	0.789
VGG-19	0.732	0.743	0.723	0.273	0.7390
Alexnets	0.804	0.783	0.784	0.229	0.806
Resnets-50	0.80	0.801	0.802	0.200	0.812
Resnets-100	0.840	0.838	0.836	0.177	0.82
Inception V3	0.678	0.677	0.675	0.675	0.681
Densenets-121	0.790	0.784	0.779	0.221	0.79
Desnsenet-119	0.777	0.781	0.78	0.229	0.774
Densenets-150	0.80	0.792	0.789	0.728	0.73
Mobilenets	0.782	0.784	0.783	0.773	0.753
SegCaps	0.87	0.92	0.910	0.09	0.910
ProposedModel	0.982	0.973	0.965	0.335	0.970

As the datasets are high, all deep learning models have shown dip in performance of prediction whereas SegCAP and Resenets-100, the proposed model has shown promising performance for increased datasets in which the proposed model has outperformed the other model in the rocketing style. Also, a similar fashion of performance as in Table 2 is observed in Table 4, which clearly shows that the proposed model has outperformed the other models. The ensemble of capsule networks and extreme learning machines has produced the best accuracy in detecting COVID-19 from the multiple sources of datasets and proves its superiority over the other algorithms.

**Table 4:** Comparative analysis of the different algorithms in detecting the COVID-19 using dataset 3.

**Algorithm**	**Performance metrics**				
	**Accuracy**	**Precision**	**Recall**	**Specificity**	**F1-Score**
VGG-16	0.8269	0.833	0.8234	0.170	0.832
VGG-19	0.833	0.843	0.823	0.173	0.840
Alexnets	0.834	0.823	0.814	0.189	0.826
Resnets-50	0.845	0.823	0.832	0.164	0.834
Resnets-100	0.849	0.843	0.834	0.167	0.838
Inception V3	0.80	0.82	0.821	0.190	0.801
Densenets-121	0.82	0.83	0.834	0.167	0.812
Desnsenet-119	0.78	0.793	0.80	0.200	0.80
Densenets-150	0.81	0.802	0.794	0.80	0.73
Mobilenets	0.782	0.784	0.778	0.783	0.778
SegCaps	0.89	0.934	0.923	0.07	0.930
ProposedModel	0.982	0.973	0.965	0.335	0.970

Finally, comparison of a proposed model with the other blockchain-based learning models is used in terms of detection accuracy, trust level, and number of datasets. Table 5 presents the comparative analysis of the blockchain-based learning model used for data sharing and data retrieval. Tables 5–7 show that the proposed model has found its good place of suitability in the blockchain for a better data sharing and retrieval process without sacrificing the data's privacy and security.

**Table 5:** Comparative analysis between the blockchain-based learning models for COVID-19 detection of diseases using dataset 1.

**References**	**Proposed model in blockchain**	**Number of cases**	**Average accuracy performance %**	**Trust level**	**Sharing and retrieval**
Parnian et al. (40)	ResNETS	High	89.5	No	No
He et al. (41)	2D-CNN	High	85.4	No	No
Rahimzadeh et al. (44)	Federated Capsule network learning	High	91	Medium	Yes
Ours	Federated Ensembled capsule networks	High	98.5	High	Yes

**Table 6:** Comparative analysis between the blockchain-based learning models for COVID-19 detection of diseases using dataset 2.

**References**	**Proposed model in blockchain**	**Number of cases**	**Average accuracy performance %**	**Trust level**	**Sharing and retrieval**
Parnian et al. (40)	ResNETS	Very High	88.4	No	No
He et al. (41)	2D-CNN	Very High	83.3	No	No
Rahimzadeh et al. (44)	Federated Capsule network learning	Very High	89	Medium	Yes
Ours	Federated Ensembled capsule networks	Very High	98.5	High	Yes

**Table 7:** Comparative analysis between the blockchain-based learning models for COVID-19 detection of diseases using dataset 3.

**References**	**Proposed model in blockchain**	**Number of cases**	**Average accuracy performance %**	**Trust level**	**Sharing and retrieval**
Parnian et al. (40)	ResNETS	High	89.5	No	No
He et al. (41)	2D-CNN	High	85.4	No	No
Rahimzadeh et al. (44)	Federated capsule network learning	High	91	Medium	Yes
Ours	Federated Ensembled capsule networks	High	98.5	High	Yes

The proposed model and federated model proposed in He et al. (41) have shown the same characteristics, but the proposed method has shown a slight edge with a 7% increment of trust. It is due to the inclusion of chaotic encryptions and detection accuracy in terms of integration of capsule and ELM.

Furthermore, the time complexity and space complexity of the proposed model are compared with the other state-of-the-art classical models. The time complexity is calculated based on big-oh notation, which is generally mentioned as O(n). Since all the classical learning models execute on the centralized system, the number of N computations increases with the power of two. The proposed model uses the distributed system, and the number of execution times decreases based on the number of nodes utilized for the training. In the experimentation, we used 5 nodes which are utilized for training, in which N is reduced to five. Table 8 represents the comparative analysis of different time complexity with the proposed and other existing classical models. Space complexity is measured by memory utilized by the algorithm.

**Table 8:** Comparative analysis between the blockchain-based model with other learning.

**References**	**Proposed model in blockchain**	**Time complexity**	**Space complexity**
Parnian et al. (40)	ResNETS	O(n^2n^)	6.92 MB
He et al. (41)	2D-CNN	O(n^2n−1^)	5.54 MB
Rahimzadeh et al. (44)	Federated capsule network learning	O(n^2n−5^)	3.25 MB
Ours	Federated ensembled capsule networks	O(n^2n−5^)	2.85 MB

The table represents the time complexity of the different algorithms. From Table 8, it is proved that the federated learning model has achieved less complexity than the other classical models due to a distributed system. The space complexity is the amount of the memory consumed by the model for better execution. The federated model consumes less memory due to its distributive nature whereas the proposed federated model with the usage of an extreme learning machine has reduced the space complexity because of the feed forward nature.

## Conclusion

The existing classical AI techniques often require centralized data storage and training for the predictive model development which leads to computational complexity and also affects privacy. To overcome the problem, this paper proposes the blockchain empowered federated framework to enhance the perception of multiple sources of heterogeneous CT images. It shares the data among the hospitals while maintaining privacy and security. Also, an ensemble of capsule networks and extreme learning machines are used for effective feature extraction and classification to detect the COVID-19 among the different sources of publicly available heterogenous CT image datasets. Furthermore, federated learning is adopted for the collaborative training of hospitals backed with blockchain technology. Also, the addition of chaotic encryption keys in the process of data retrieval and sharing process has added more trust in terms of maintaining privacy and security. Comprehensive experimentation has been conducted and compared with the other deep learning algorithms. Additionally, the performance of the proposed algorithm has been compared with the other blockchain powered federated deep learning modules. The results demonstrate that the proposed model has shown the highest performance in terms of accuracy, precision, recall, and high F1-score and proves to be more vital than the other algorithms in terms of trust and datasets. Though the proposed method has shown better performance, it needs improvisation in handling the real-time clinical databases. In the future, an effort will be done to reduce the latency of the blockchain and optimize the cost-effectiveness of the solution.

## Data Availability Statement

The original contributions presented in the study are included in the article/supplementary material, further inquiries can be directed to the corresponding author.

## Ethics Statement

Ethical review and approval was not required for the study on human participants in accordance with the local legislation and institutional requirements. Written informed consent for participation was not required for this study in accordance with the national legislation and the institutional requirements.

## Author Contributions

RD contributed to the conception and design of the study. All authors contributed to manuscript revision, read, and approved the submitted version.

## Conflict of Interest

The authors declare that the research was conducted in the absence of any commercial or financial relationships that could be construed as a potential conflict of interest.

## Publisher's Note

All claims expressed in this article are solely those of the authors and do not necessarily represent those of their affiliated organizations, or those of the publisher, the editors and the reviewers. Any product that may be evaluated in this article, or claim that may be made by its manufacturer, is not guaranteed or endorsed by the publisher.
